# Supplementary material for: A hybrid-frequency programmable synthetic-dimension simulator with rich coupling on a single chip
Source: Light Sci Appl. 2026 Apr 24;15:213. doi: 10.1038/s41377-026-02309-2 (PMC13109359; doi:10.1038/s41377-026-02309-2)
Supplement: Supplementary file 1 — Supplementary Information [file 41377_2026_2309_MOESM1_ESM.pdf]

Supplementary Information for
A hybrid-frequency programmable synthetic-dimension simulator with rich coupling on a single chip

Xiao-Dong Zeng,[1,2,3,†] Zhao-An Wang,[1,2,3,4,5,†] Jia-Ming Ren,[1,2,3,†] Yi-Tao Wang,[1,2,3,*] Chun Ao,[1,2,3] Wei Liu,[1,2,3] Nai-Jie Guo,[1,2,3,4] Lin-Ke Xie,[1,2,3] Jun-You Liu,[1,2,3,4] Yu-Hang Ma,[1,2,3] Ya-Qi Wu,[1,2,3] Shuang Wang,[1,2,3,4] Pei-Yun Li,[1,2,3] Mu Yang,[1,2,3] Jin-Shi Xu,[1,2,3,4] Xi-Wang Luo,[1,2,3,4] Jian-Shun Tang,[1,2,3,4,*] Chuan-Feng Li,[1,2,3,4,*] and Guang-Can Guo[1,2,3,4]

[1] *Laboratory of Quantum Information, University of Science and Technology of China, Hefei 230026, China*
[2] *Anhui Province Key Laboratory of Quantum Network, University of Science and Technology of China, Hefei 230026, China*
[3] *CAS Center For Excellence in Quantum Information and Quantum Physics, University of Science and Technology of China, Hefei 230026, China*
[4] *Hefei National Laboratory, University of Science and Technology of China, Hefei 230088, China*
[5] *Quantum Science Center of Guangdong-Hong Kong-Macao Greater Bay Area, Shenzhen 518045, China*

(Dated: April 7, 2026)

CONTENTS

I. Experimental Chip 2

II. Theoretical Details 3
A. Deriving band structures from MZI-assisted two-resonator dynamics 3
B. Deriving the band structure of the Su-Schrieffer-Heeger model 5

III. Supplement Experimental Data 6
A. Supplementary experimental band structure results 6
B. The Creutz ladder that does not form the Aharonov-Bohm cage 7

IV. Applications of Topological Flat Band in Sideband Engineering 8

V. Effect of Modulation Strength 9
A. Effective regime for model construction (influence of modulation strength on the SSH model) 9
B. Effect of modulation strength on the construction of the Creutz ladder model 10

VI. The Effect of Photonic Molecule Resonance Mode Alignment on the Experiment 11

VII. Robustness of SSH Lattice Construction to Frequency Detuning 12
A. Regime where the modulation strength Is much smaller than the loss γ 12
B. Breakdown of the synthetic frequency lattice at large lattice constants 12

VIII. Impact of Ring Resonator Loss and RF Modulation Strength on the Measured Band Gap of the SSH Model 13

IX. The Construction of the extend SSH Model 14

† These authors contributed equally to this work.
* Corresponding authors: yitao@ustc.edu.cn, tjs@ustc.edu.cn, cfli@ustc.edu.cn

X. Quality Factor of the Resonators 15

References 16

I. EXPERIMENTAL CHIP

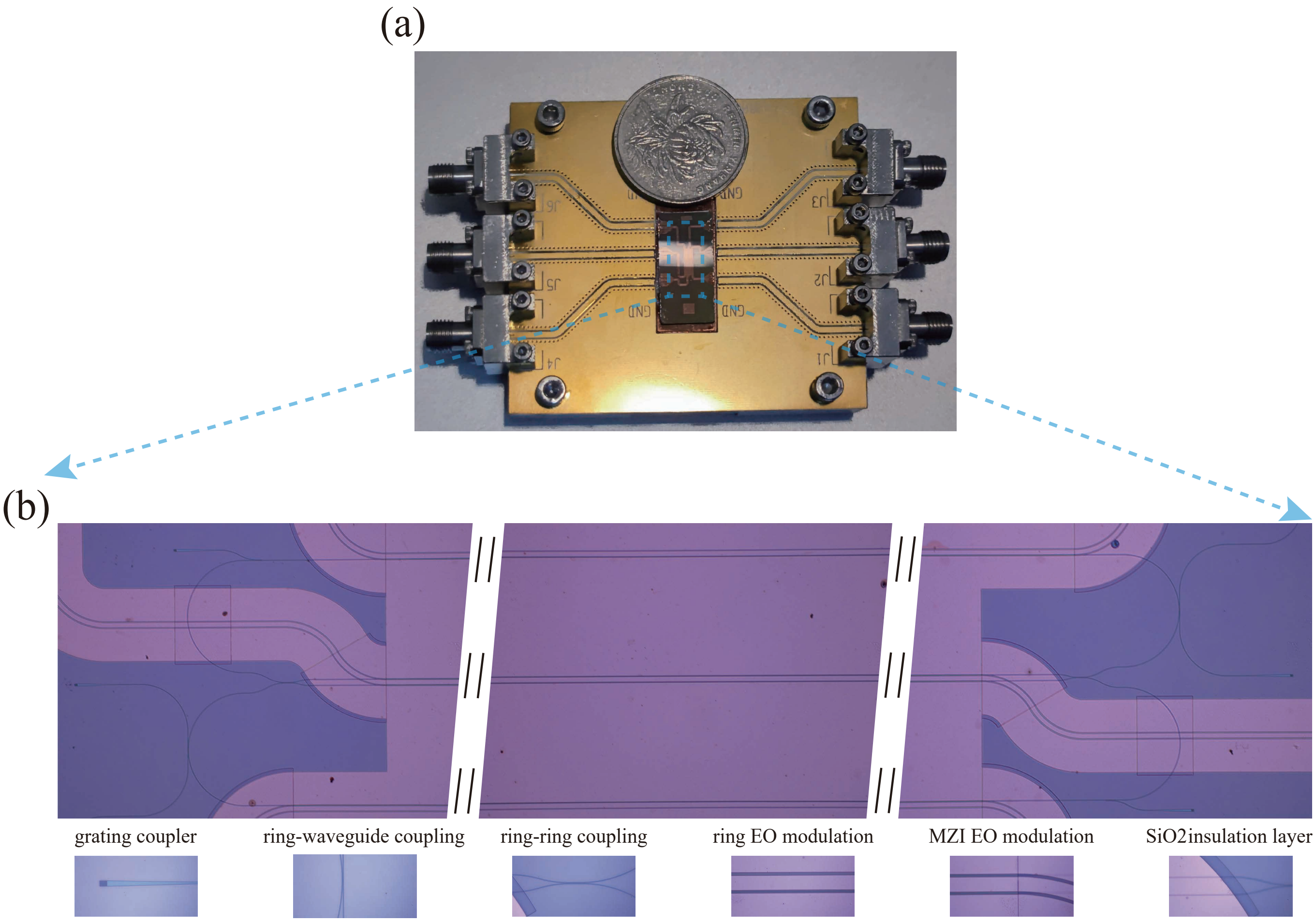

Fig. S1. **Experimental device.** (**a**) Image of the thin film lithium niobate (TFLN) photonic chip. (**b**) The optical microscope images of the device consisting of LN racetrack microresonator and electrodes. The bottom insets showcase the key devices fabricated on TFLN.

First, we design and fabricate the double resonators coupled by an MZI with three sets of electrodes on the TFLN platform. The fabricated chip is shown in Fig. S1a. The size of the entire chip is 10 mm $\times$ 20 mm, similar size to a coin. Fig. S1b presents the optical microscope image of the racetrack microresonator. The illustration at the bottom of Fig. S1b shows the images of the fabricated on-chip optical devices and electrodes, such as grating couplers, MZI modulation electrodes, etc.

II. THEORETICAL DETAILS

A. Deriving band structures from MZI-assisted two-resonator dynamics

In this subsection, we present the theoretical details of constructing the frequency lattice using a single resonant peak, under the condition of spatially tunable coupling between two-ring resonator. Based on our previous work [1], we have provided a theoretical analysis of the method for constructing a frequency lattice using intra-resonant modes in a single resonator, and a similar approach is employed here. We let a be the annihilation operator of the ring-A resonant peak with central frequency ω_0 and a_n be the annihilation operator the n-th mode with frequency $n\Omega$. Similarly, for ring-B resonator, we define the corresponding annihilation b and b_n [2, 3]. For a and b, they satisfy $a = \frac{1}{\sqrt{N_t}}\sum_{n=-N}^{N} a_n(t)e^{-in\Omega t}$, $b = \frac{1}{\sqrt{N_t}}\sum_{n=-N}^{N} b_n(t)e^{-in\Omega t}$. We let the light be injected from Resonator A and the input-output relation can be written as:

$$
\begin{aligned}
\partial_t \sum_{n=-N}^{N} \frac{1}{\sqrt{N_t}} a_n e^{-in\Omega t} &= \sum_{n=-N}^{N} (-i\omega_0 - \gamma/2) \frac{1}{\sqrt{N_t}} a_n e^{-in\Omega t} + iJ_B^H \sum_{n=-N}^{N} \frac{1}{\sqrt{N_t}} a_n e^{-in\Omega t} + iJ^V \sum_{n=-N}^{N} \frac{1}{\sqrt{N_t}} b_n e^{-in\Omega t} \\
&\quad + iJ^C \sum_{n=-N}^{N} \frac{1}{\sqrt{N_t}} b_{n+1} e^{-i(n+1)\Omega t} + i\sqrt{\gamma_c}\, s_p e^{-i\omega_p t}, \\
\partial_t \sum_{n=-N}^{N} \frac{1}{\sqrt{N_t}} b_n e^{-in\Omega t} &= \sum_{n=-N}^{N} (-i\omega_0 - \gamma/2) \frac{1}{\sqrt{N_t}} b_n e^{-in\Omega t} + iJ_A^H \sum_{n=-N}^{N} \frac{1}{\sqrt{N_t}} b_n e^{-in\Omega t} + iJ^V \sum_{n=-N}^{N} \frac{1}{\sqrt{N_t}} a_n e^{-in\Omega t} \\
&\quad + iJ^C \sum_{n=-N}^{N} \frac{1}{\sqrt{N_t}} a_{n+1} e^{-i(n+1)\Omega t}, \\
\sum_{n=-N}^{N} \frac{1}{\sqrt{N_t}} \dot{a}_n e^{-in\Omega t} &= \sum_{n=-N}^{N} (-i\omega_0 + in\Omega - \gamma/2) \frac{1}{\sqrt{N_t}} a_n e^{-in\Omega t} + iJ_A^H \sum_{n=-N}^{N} \frac{1}{\sqrt{N_t}} a_n e^{-in\Omega t} + iJ^V \sum_{n=-N}^{N} \frac{1}{\sqrt{N_t}} b_n e^{-in\Omega t} \\
&\quad + iJ^C \sum_{n=-N}^{N} \frac{1}{\sqrt{N_t}} b_{n+1} e^{-i(n+1)\Omega t} + i\sqrt{\gamma_c} s_p e^{-i\omega_p t}, \\
\sum_{n=-N}^{N} \frac{1}{\sqrt{N_t}} \dot{b}_n e^{-in\Omega t} &= \sum_{n=-N}^{N} (-i\omega_0 + in\Omega - \gamma/2) \frac{1}{\sqrt{N_t}} b_n e^{-in\Omega t} + iJ_B^H \sum_{n=-N}^{N} \frac{1}{\sqrt{N_t}} b_n e^{-in\Omega t} + iJ^V \sum_{n=-N}^{N} \frac{1}{\sqrt{N_t}} a_n e^{-in\Omega t} \\
&\quad + iJ^C \sum_{n=-N}^{N} \frac{1}{\sqrt{N_t}} a_{n+1} e^{-i(n+1)\Omega t}.
\end{aligned}
\tag{S1}
$$

where $\gamma = \gamma_c + \gamma_{in}$ is the total decay rate which is the sum of the resonator-waveguide coupling rate and the intrinsic loss rate of the rensonator. $J_A^H (J_B^H)$, J^V and J^C represent the horizontal, vertical, and cross-coupling strengths in the model, respectively. Ω is the modulation frequency, and s_p and ω_p are the amplitude and frequency of the probing laser. For the case that $\Omega \ll \gamma$ and J, the term $in\Omega$ in Eq. (S1) can be neglected. We consider the nearest-neighbor coupling in our demonstration ($J_A^H(t) = 2J^H \cos(\Omega t + \phi_A^H)$, $J_B^H(t) = 2J^H \cos(\Omega t + \phi_B^H)$, $J^C(t) = 2J^C \cos(\Omega t + \phi_C)$, J^V =constant), and the analytical treatment of long-range coupling scenarios proceeds similarly. The large number $N_t = 2N + 1$ is the total number of lattice points. By transforming $a_n e^{i\omega_p t} \to a_n$ and denoting the detuning of the

probing laser as $\Delta\omega = -\omega_0 - \omega_p$, we have:

$$
\begin{aligned}
\sum_n \dot{a}_n e^{-in\Omega t} &= (i\Delta\omega - \gamma/2)\sum_n a_n e^{-in\Omega t} + iJ^H(\sum_n a_n e^{-i(n+1)\Omega t - i\phi_A^H} + \sum_n a_n e^{-i(n-1)\Omega t + i\phi_A^H}) + iJ^V \sum_n b_n e^{-in\Omega t} \\
&+ iJ^C(\sum_n b_n e^{-i(n+1)\Omega t - i\phi_C} + \sum_n b_n e^{-i(n-1)\Omega t} + \phi_C) + i\sqrt{N_t}\sqrt{\gamma_c}s_p, \\
\sum_n \dot{b}_n e^{-in\Omega t} &= (i\Delta\omega - \gamma/2)\sum_n b_n e^{-in\Omega t} + iJ^H(\sum_n b_n e^{-i(n+1)\Omega t - i\phi_B^H} + \sum_n b_n e^{-i(n-1)\Omega t + i\phi_B^H}) + iJ^V \sum_n a_n e^{-in\Omega t} \\
&+ iJ^C(\sum_n a_n e^{-i(n+1)\Omega t - i\phi_C} + \sum_n a_n e^{-i(n-1)\Omega t + i\phi_C}).
\end{aligned}
\tag{S2}
$$

The equation can be solved by extracting the coefficients of the $e^{-in\Omega t}$ terms with respect to n.

$$
\begin{aligned}
\dot{a}_n &= (i\Delta\omega - \gamma/2)a_n + iJ^H(a_{n-1}e^{-i\phi_A^H} + a_{n+1}e^{i\phi_A^H}) + iJ^V b_n + iJ^C(b_{n-1}e^{-i\phi_C} + b_{n+1}e^{i\phi_C}) + i\sqrt{N_t}\sqrt{\gamma_c}s_p e^{in\Omega t}\delta_{n,0}, \\
\dot{b}_n &= (i\Delta\omega - \gamma/2)b_n + iJ^H(b_{n-1}e^{-i\phi_B^H} + b_{n+1}e^{i\phi_B^H}) + iJ^V a_n + iJ^C(a_{n-1}e^{-i\phi_C} + a_{n+1}e^{i\phi_C}).
\end{aligned}
\tag{S3}
$$

Eq. (S3) can be rewritten in the quasi-momentum space by using transformation $a_k = \frac{1}{\sqrt{N_t}}\sum_n a_n e^{-in\Omega k}$, $b_k = \frac{1}{\sqrt{N_t}}\sum_n b_n e^{-in\Omega k}$, we have

$$
\begin{aligned}
i\dot{a}_k &= (-\Delta\omega - i\gamma/2 - 2J^H\cos(k\Omega + \phi_A^H))a_k - J^V b_k - 2J^C\cos(k\Omega + \phi_C)b_k + i\sqrt{\gamma_c}s_p, \\
i\dot{b}_k &= (-\Delta\omega - i\gamma/2 - 2J^H\cos(k\Omega + \phi_B^H))b_k - J^V a_k - 2J^C\cos(k\Omega + \phi_C)a_k.
\end{aligned}
\tag{S4}
$$

Here we define $|\varphi_k\rangle = (a_k, b_k)^{\mathrm{T}}$, $|0\rangle = (1,0)^{\mathrm{T}}$ and $|1\rangle = (0,1)^{\mathrm{T}}$. The above equation can be written as:

$$
-\left[\Delta\omega + i\gamma/2 - H_k(t) + i\partial_t\right]|\varphi_k\rangle = \sqrt{\gamma_c}T\delta(t-k)s_p\,|0\rangle\,. \tag{S5}
$$

The Hamiltonian in the k-space is:

$$
H_k = -2\begin{bmatrix} J^H\cos(k\Omega + \phi_A^H) & J^V/2 + J^C\cos(k\Omega + \phi_C) \\ J^V/2 + J^C\cos(k\Omega + \phi_C) & J^H\cos(k\Omega + \phi_B^H) \end{bmatrix}. \tag{S6}
$$

This exactly corresponds to the k-space Hamiltonian of the Creutz ladder. When $t = k$ is sufficiently larger than $1/\gamma$, the system evolves to a steady state, and the steady-state solution of Eq. (S4) can be obtained. Let ψ_i be the eigenstate of H_k, then:

$$
\langle\psi_i\,|\Delta\omega + i\gamma/2 - H_k|\,\varphi_k\rangle = -\sqrt{\gamma_c}s_p\langle\psi_i|0\rangle. \tag{S7}
$$

Let E_i be the eigenvalue of H_k, then:

$$
(\Delta\omega + i\gamma/2 - E_i)\,\langle\psi_i\mid\varphi_k\rangle = -\sqrt{\gamma_c}s_p\,\langle\psi_i\mid 0\rangle\,. \tag{S8}
$$

So we can obtain:

$$
\langle\psi_i|\varphi_k\rangle = \frac{-\sqrt{\gamma_c}s_p\,\langle\psi_i|0\rangle}{\Delta\omega + i\gamma/2 - E_i} \tag{S9}
$$

Thus, we can obtain the expression of a_k:

$$a_k = \langle 0 \mid \varphi_k \rangle = \langle 0 \mid \psi_1 \rangle \langle \psi_1 \mid \varphi_k \rangle + \langle 0 \mid \psi_2 \rangle \langle \psi_2 \mid \varphi_k \rangle = -\left(\frac{\sqrt{\gamma_c} s_p \left|\langle 0 \mid \psi_1 \rangle\right|^2}{\Delta\omega + i\gamma/2 - E_1} + \frac{\sqrt{\gamma_c} s_p \left|\langle 0 \mid \psi_2 \rangle\right|^2}{\Delta\omega + i\gamma/2 - E_2} \right). \tag{S10}$$

Now we obtain the expression for transmittance as follows:

$$T_{out} = \left| \frac{1}{s_p} e^{i\omega_p t} i\sqrt{\gamma_c} \sum_n \frac{1}{\sqrt{N_t}} a_n e^{-in\Omega t} \right|^2 = \left| \frac{i\sqrt{\gamma_c} a_k|_{k=t}}{s_p} \right|^2 \tag{S11}$$

.

Eq. (S11) indicates that the probing laser excites one slice of the band structure.

B. Deriving the band structure of the Su-Schrieffer-Heeger model

In this subsection, we will explain how to construct the Su-Schrieffer-Heeger (SSH) model using our approach. In the previous subsection, we always align the mode frequencies of the two resonators. However, we can intentionally shift them by δ ($\delta < \Omega_R/2$) through a DC signal applied to the resonator. As shown in Fig. 4A in the main text, J^V is changed to a modulation applied to the MZI with frequency δ, while J^C transforms into modulations with frequencies $\Omega_R + \delta$ and $\Omega_R - \delta$ applied to the MZI. We integrate this method with the intra-resonant frequency-lattice scheme. Considering two resonators with a center-frequency difference of δ and employed with two modulation frequencies $\delta - \Omega$ and δ ($\Omega \ll \gamma$), we have

$$\begin{aligned} \frac{1}{\sqrt{N_t}} \sum_n \dot{a}_n e^{-in\Omega t} &= (-i\omega_0 + in\Omega - \gamma/2) \frac{1}{\sqrt{N_t}} \sum_n a_n e^{-in\Omega t} + i(2J_1^C \cos(\delta t) + 2J_2^C \cos((\delta - \Omega)t)) \sum_n \frac{1}{\sqrt{N_t}} b_n e^{-in\Omega t} + i\sqrt{\gamma_c} s_p e^{-i\omega} \text{[illegible]} \\ \frac{1}{\sqrt{N_t}} \sum_n \dot{b}_n e^{-in\Omega t} &= (-i(\omega_0 + \delta) + in\Omega - \gamma/2) \frac{1}{\sqrt{N_t}} \sum_n b_n e^{-in\Omega t} + i(2J_1^C \cos(\delta t) + 2J_2^C \cos((\delta - \Omega)t)) \sum_n \frac{1}{\sqrt{N_t}} a_n e^{-in\Omega t}. \end{aligned} \tag{S12}$$

Here, a_n and b_n are the intra-resonant frequency modes of the two resonators, J_1^C and J_2^C represent the modulation amplitudes of the two modulations applied to the MZI, and the definitions of other parameters remain consistent with those in Subsection A. Following a similar approach, we perform the transformation $a_n e^{i\omega_p t} \to a_n$ and $b_n e^{i(\omega_p + \delta)} \to b_n$, define $\Delta\omega = -\omega_0 - \omega_p$, and neglect the $in\Omega$ term. After simplification, we obtain

$$\begin{aligned} \sum_n \dot{a}_n e^{-in\Omega t} &= (-i\Delta\omega - \gamma/2) \sum_n a_n e^{-in\Omega t} + i(2J_1^C \cos(\delta t) + 2J_2^C \cos((\delta - \Omega)t)) \sum_n b_n e^{-i(n\Omega + \delta)t} + i\sqrt{\gamma_c N_t} s_p, \\ \sum_n \dot{b}_n e^{-in\Omega t} &= (-i\Delta\omega - \gamma/2) \sum_n b_n e^{-in\Omega t} + i(2J_1^C \cos(\delta t) + 2J_2^C \cos((\delta - \Omega)t)) \sum_n a_n e^{-i(n\Omega - \delta)t}. \end{aligned} \tag{S13}$$

Further applying the rotating wave approximation, we retain only the terms without δ in the second summation on the right-hand side of the two equations (terms containing δ correspond to the modes jumping to the frequencies that are unsupported by the two resonators, and are effectively reflected due to mode mismatch). After simplification, we obtain

$$\begin{aligned} \sum_n \dot{a}_n e^{-in\Omega t} &= (-i\Delta\omega - \gamma/2) \sum_n a_n e^{-in\Omega t} + i \sum_n ((J_1^C b_n + J_2^C b_{n-1}) e^{-in\Omega t} + i\sqrt{\gamma_c N_t} s_p, \\ \sum_n \dot{b}_n e^{-in\Omega t} &= (-i\Delta\omega - \gamma/2) \sum_n b_n e^{-in\Omega t} + i \sum_n (J_1^C a_n + J_2^C a_{n+1}) e^{-in\Omega t}. \end{aligned} \tag{S14}$$

Solving the equations term by term

$$\begin{aligned} \dot{a}_n &= (-i\Delta\omega - \gamma/2) a_n + i(J_1^C b_n + J_2^C b_{n-1}) + i\sqrt{\gamma_c N_t} s_p e^{in\Omega t}, \\ \dot{b}_n &= (-i\Delta\omega - \gamma/2) b_n + i(J_1^C a_n + J_2^C a_{n+1}), \end{aligned} \tag{S15}$$

and rewritting them in the quasi-momentum space using transformation $a_k = \frac{1}{\sqrt{N_t}}\sum_n a_n e^{-in\Omega k}$, $b_k = \frac{1}{\sqrt{N_t}}\sum_n b_n e^{-in\Omega k}$, we have

$$\begin{aligned} \dot{a}_k &= (-i\Delta\omega - \gamma/2)a_k + iJ_1^C b_k + iJ_2^C b_k e^{i\Omega k} + \sqrt{\gamma_c}s_p, \\ \dot{b}_k &= (-i\Delta\omega - \gamma/2)b_k + iJ_1^C a_k + iJ_2^C a_k e^{-i\Omega k}. \end{aligned} \quad \text{(S16)}$$

Similarly, we define $|\varphi_k\rangle = (a_k, b_k)^{\mathrm{T}}$, $|0\rangle = (1,0)^{\mathrm{T}}$ and $|1\rangle = (0,1)^{\mathrm{T}}$. The above equations can be written as:

$$-\left[\Delta\omega + i\gamma/2 - H_k(t) + i\partial_t\right]|\varphi_k\rangle = \sqrt{\gamma_c}T\delta(t-k)s_p\,|0\rangle\,. \quad \text{(S17)}$$

The Hamiltonian in k-space is

$$H_k = \begin{bmatrix} 0 & J_1^C + J_2^C e^{i\Omega k} \\ J_1^C + J_2^C e^{-i\Omega k} & 0 \end{bmatrix}, \quad \text{(S18)}$$

Similar to the previous section, we can obtain the expressions for a_k and T_{out}.

where $G = |G|\,e^{i\varphi(k_f)} = J_1^C + J_2^C e^{ik_f\Omega + i\phi}$ and $\varphi(k_f) = \mathrm{Arg}(G)$ is the argument of G. Here, k_f denotes the wave vector reciprocal to the frequency dimension. The topological properties of the SSH model can be described by the Zak phase:

$$\varphi_{Zak} = \frac{1}{2}\int_{-\pi}^{\pi}\frac{\partial\varphi(k_f)}{\partial k_f}\,dk_f. \quad \text{(S19)}$$

The Zak phase takes two values, which are $\varphi_{Zak} = \pi$ for the topologically non-trivial case ($J_1^C < J_2^C$) and $\varphi_{Zak} = 0$ for the trivial case ($J_1^C > J_2^C$). The expression of the band-structure theoretical solution for the SSH model is as follows:

$$\epsilon_{k_f,m} = m\sqrt{(J_1^C)^2 + (J_2^C)^2 + 2J_1^C J_2^C\cos(k_f\Omega + \phi)}. \quad \text{(S20)}$$

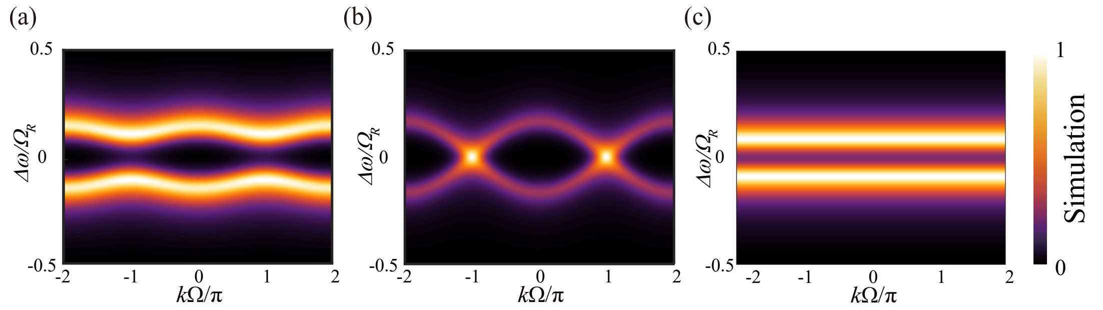

Fig. S2. **The band structure diagram is obtained from Eq. (S18) into Eq. (S11).** The band structure of the SSH model, obtained by solving the T_{out} equation derived from the coupled-mode theory, is varied via the adjustments of the parameters of J_1^C and J_2^C. Plot a-c respectively demonstrate three configurations: $J_2^C = 0.12\Omega > J_1^C = 0.02\Omega$; $J_1^C = J_2^C = 0.08\Omega$; $J_1^C = 0.08\Omega$, $J_2^C = 0$.

III. SUPPLEMENT EXPERIMENTAL DATA

A. Supplementary experimental band structure results

In this section, we present more experimental band structure data, including those from Hall ladder and Creutz ladder. In Fig. S3, we show the diverse band structures of the Hall ladder under different effective magnetic fluxes, and we also present the changes in the band structures under varying modulation strengths. In Figs. S4 and S5, we present the diverse band structures of the Creutz ladder model under different effective magnetic fluxes.

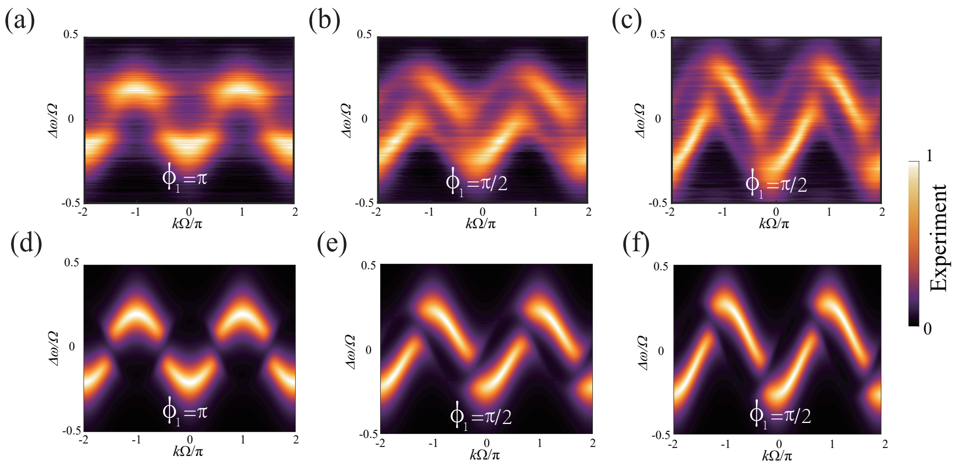

Fig. S3. **Hall ladder band structure with different effective magnetic flux (ϕ_1) and coupling strengths.** (**a**) The heat maps are the experimentally obtained band structures of Hall ladder with the coupling strength $r = J^V/2J^H = 0.8$, $J_H = 0.085\Omega_R$, $\Omega = 2\pi \times 10$ MHz, and the effective magnetic flux $\phi_1 = \pi$. (**b, c**) Band structures for effective magnetic flux $\phi_1 = \pi/2$ with couplings $r = 0.8$ and $r = 0.5$ ($J_H = 0.125\Omega_R$, $\Omega = 2\pi \times 10$ MHz). (**d-f**) Plots d-f show the numerically calculated band structure corresponding to the experimental data in the upper panels.

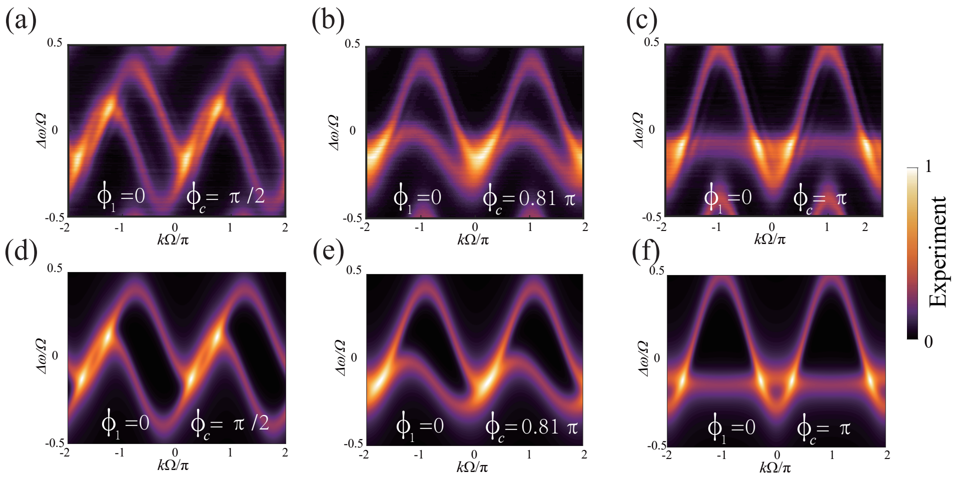

Fig. S4. **The heat maps display the band structure of the Creutz ladder with different ϕ_1 and ϕ_c.** (**a-c**) The coupling strengths are $J^V/2J^H = 0.6$, $J^C/J^H = 0.48$ and $J^H = 0.125\Omega_R$. The values of (ϕ_1, ϕ_C) corresponding to Plots a-c are (0, $\pi/2$), (0, 0.81π) and (0, π), respectively. (**d-f**) Plots d-f show the numerically calculated band structure corresponding to the experimental data in the upper panels.

B. The Creutz ladder that does not form the Aharonov-Bohm cage

In order to better show the strong mode locality brought by the topological flat band, as a comparison, control experiments with zero magnetic flux ($\phi_A^H = \phi_B^H = 0$, $\phi_C = 0$, shown in the Fig. S6a) show delocalized mode distributions, confirming the absence of cage under non-topological conditions. Fig. S6b shows the band structure at this time, with obvious band crossing and no localization of photon states. Since there is no Aharonov-Bohm (AB) cage effect, namely, no constraint effect, the modes are spread out and there are higher-order frequency modes (Figs. S6c, d).

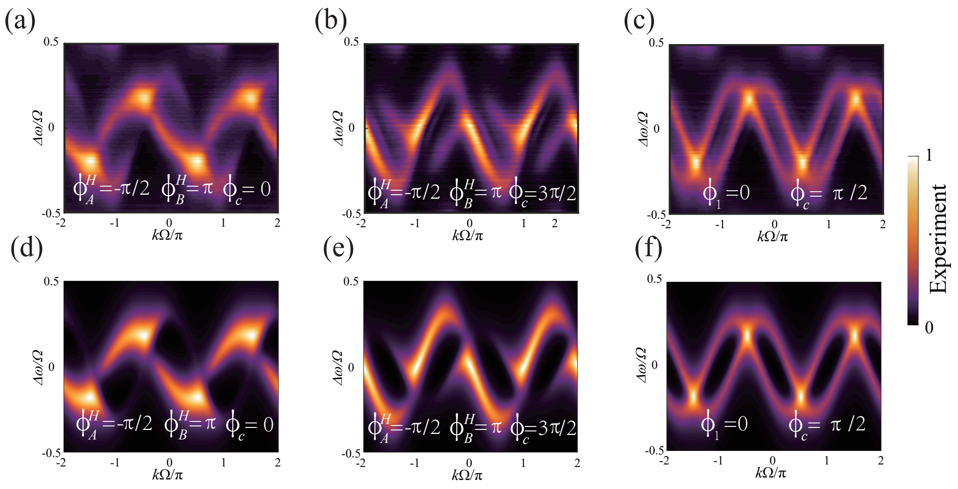

Fig. S5. **The heat maps display the band structures of the Creutz ladder with $J^V = 0$ and different ϕ_A, ϕ_B, ϕ_C (and ϕ_1).** (**a-c**) Other coupling strengths are $J^C/J^H = 1$ and $J^H = 0.11\Omega_R$. The values of (ϕ_A^H, ϕ_B^H, ϕ_C) corresponding to Plots a-c are (-$\pi/2$, π, 0), (-$\pi/2$, π, $3\pi/2$) and (0, 0, $\pi/2$). (**d-f**) Plots d-f show the numerically calculated band structure corresponding to the experimental data in the upper panels.

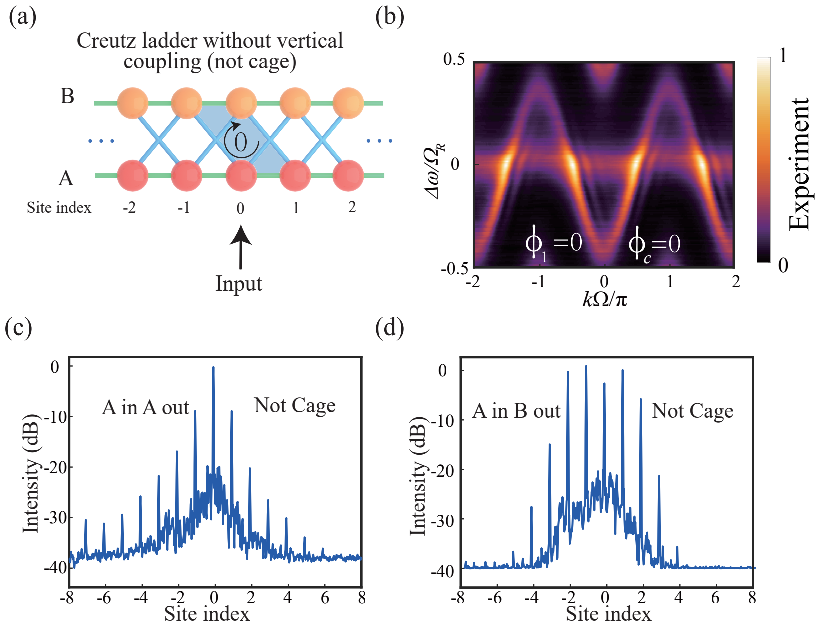

Fig. S6. **Mode distribution in the absence of AB cage effect.** (**a**) Illustration of the not-caged situation where the phase collected in the blue area is zero. The light normally spreads out along the frequency axis. (**b**) The band structure that is not the AB cage case ($\phi_A^H = \phi_B^H = 0$, $\phi_C = 0$). (**c,d**) The experimental results of the not-caged situation, where more modes survive compared to the caged situation.

IV. APPLICATIONS OF TOPOLOGICAL FLAT BAND IN SIDEBAND ENGINEERING

By implementing the modulated Creutz ladder lattice in the main text, the photon localization phenomenon (i.e., AB cage effect) at the topological flat band can be applied to the on-demand sideband design. Owing to the flexible construction of the intra-resonant frequency lattice, the continuous and precise sideband modulation becomes achievable, as demonstrated in Fig. S7. This rapid (depending on the electro-optic modulation rate) and deterministic

optical-frequency mode conversion retains only the first-order sidebands, and the spectral structure of this localized comb is extremely simple and symmetric. In the future, this approach may have a significant impact on microwave signal generation, optical communications, precision measurements, and related fields. For example, these simple and precisely tunable frequency peaks make spectral calibration and signal processing much easier, thereby enabling high-precision measurement.

As shown in Fig. S7a, the obtained AB cage results demonstrate excellent robustness across various Ω configurations. With increasing Ω magnitude, however, the number of lattice sites in the intra-resonant lattice progressively decreases, leading to gradual degradation of the lattice system's structural integrity. Remarkably, the system maintains superior sideband tunability within the 300-MHz operational window (including a 100-MHz range with high degree of suppression of the non-demand frequencies). As shown in Fig. S7b, when the Creutz ladder model is constructed using intra-resonant frequency lattice sites and the AB cage effect is realized, the effect demonstrates notable robustness to laser detuning. This indicates that laser detuning has minimal impact on the manifestation of the AB cage effect.

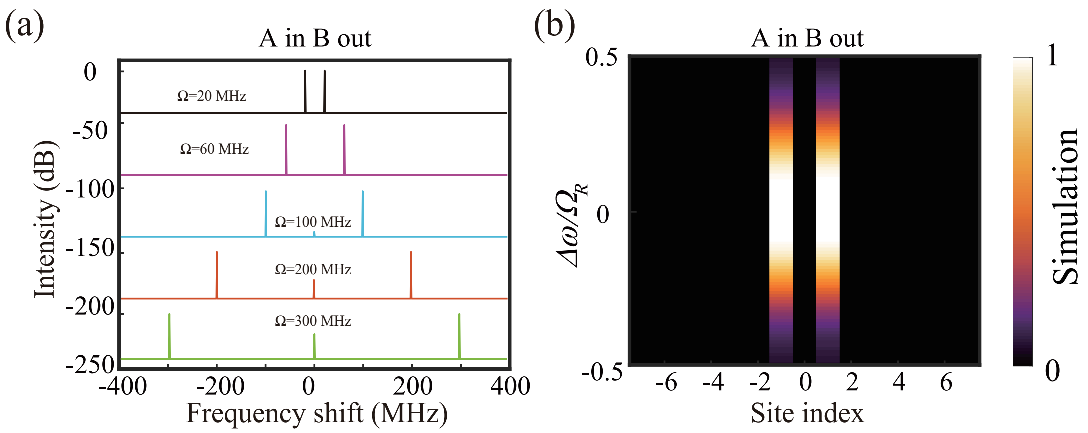

Fig. S7. **Robustness and tunability in the sideband engineering.** (**a**) Simulation of the mode distribution from the drop port of Resonator B when different RF signals are applied in the AB cage situation. (**b**) Mode distribution across the intra-resonant frequency lattice as a function of the probe-laser detuning $\Delta\omega$, measured at the drop port of Resonator B, during the emergence of the AB cage effect.

V. EFFECT OF MODULATION STRENGTH

A. Effective regime for model construction (influence of modulation strength on the SSH model)

Attention should be paid to the fact that constructing the asymmetric coupling lattice via intra-resonant frequency lattice in our device requires control of the modulation strength. Notably, despite the initial detuning of the resonator modes ($\delta > \gamma$) has suppressed the coupling between the two resonators to some extent, when excessive modulation strength is applied, the density of state (DOS) are broadened, which leads to unintended mode overlap. This phenomenon ultimately compromises the well-defined characteristics of the SSH lattice configuration. As shown in Fig. S8, the emergence of non-negligible noise within the non-band regions and the progressive degradation of the SSH-band-structure resolution are observed with increasing modulation strength. This phenomenon just originates from the mode overlap of these two resonators.

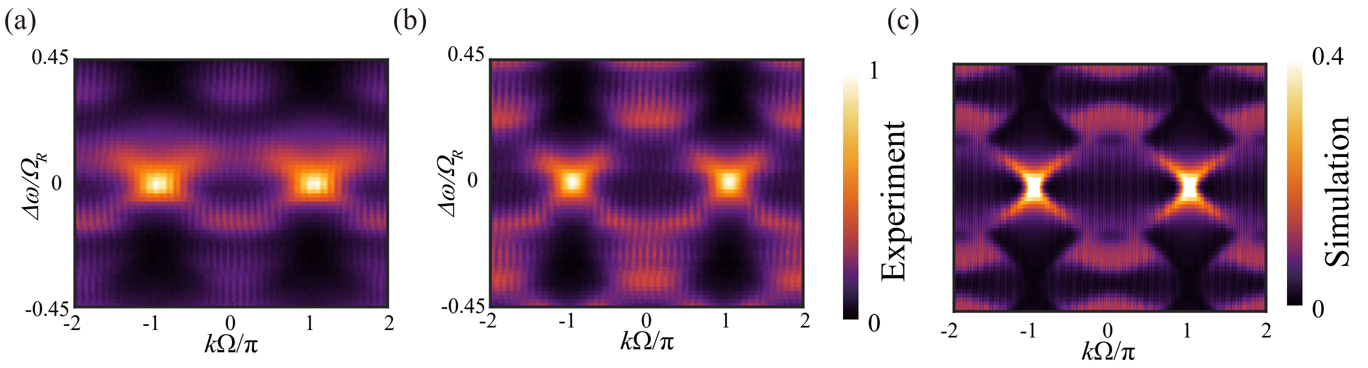

Fig. S8. **The impact of excessive modulation strength on the construction of the SSH model.** (**a**) Experimentally-measured stacked transmission spectra with modulation strength set at -9 dBm. (**b**) Experimentally-measured stacked transmission spectra with modulation strength set at -3 dBm. (**c**) Simulated stacked transmission spectra using photonic simulating software with modulation strength $V_{pp} = 20$ V.

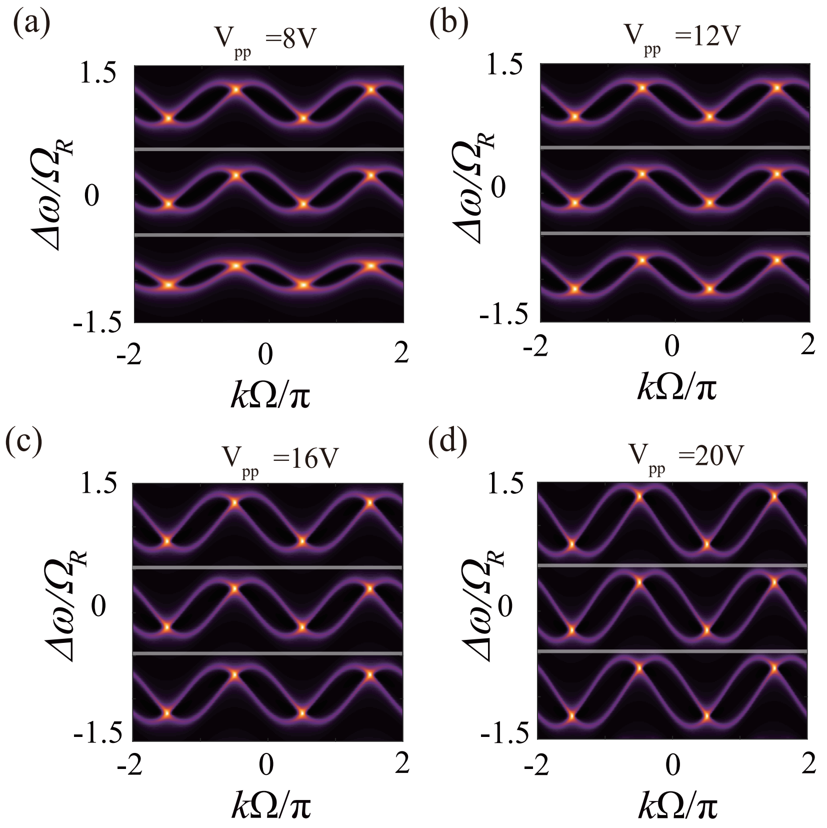

Fig. S9. **Evolution of the typical band structure of the Creutz ladder model (with the same phase conditions as in Fig. S5c) as a function of modulation strength.** (**a-d**): The band structure evolution as the modulation strength increases from $V_{\mathrm{pp}} = 8$ V to $V_{\mathrm{pp}} = 20$ V

B. Effect of modulation strength on the construction of the Creutz ladder model

In symmetric coupling models such as the Hall ladder or Creutz ladder, the resonator modes are naturally aligned, so increasing the modulation strength mainly enhances intra-resonant site coupling without introducing detuning [4–6]. As a result, these models tolerate modulation-induced broadening better than detuned models like SSH. Fig.

S9 shows the evolution of the Creutz ladder bands with increasing modulation strength, which remain clearly visible.

VI. THE EFFECT OF PHOTONIC MOLECULE RESONANCE MODE ALIGNMENT ON THE EXPERIMENT

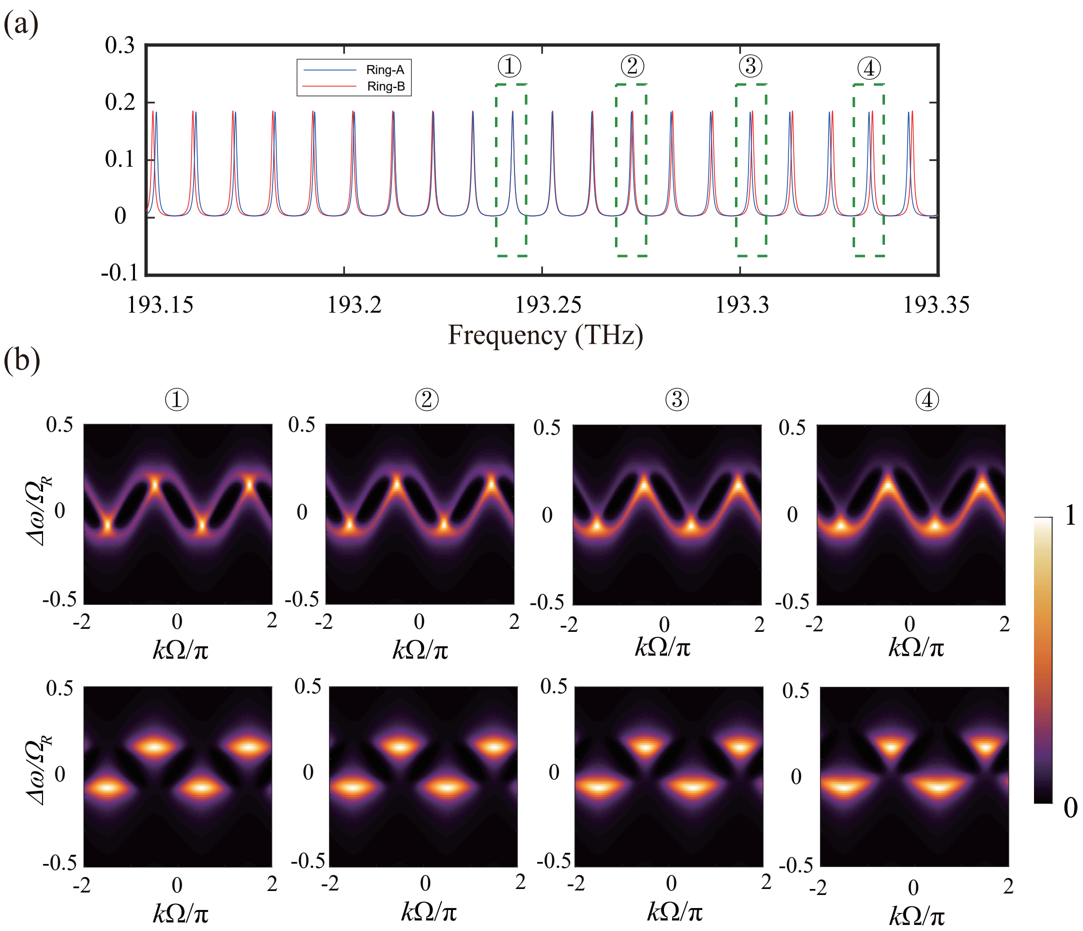

Fig. S10. **The effect of mode misalignment in photonic molecule resonators, caused by differences in ring length, on the simulated model.** (**a**) Transmission spectra of two lithium niobate resonators with different ring lengths, where ring A has an FSR of 10 GHz and ring B has an FSR of 10.1 GHz. The resonance modes of the photonic molecule are aligned at position ①. (**b**) Impact of the photonic molecule resonance detuning, as shown in Fig. a, on the band structure of the Creutz ladder model. The upper row corresponds to the model parameters of $J^V = 0, J^H = J^C, \phi_1 = 0, \phi_C = \pi/2$, while the lower row shows the model parameters corresponding to the topological flat-band regime ($J^V = 0, J^H = J^C, \phi_1 = \pi, \phi_C = \pi/2$).

In the present architecture, the synthetic frequency lattice is constructed using a single longitudinal mode from each resonator forming the photonic molecule. As long as the central resonance frequencies of the two resonators are aligned, differences in their FSRs do not enter the effective Hamiltonian and therefore do not affect the simulated dynamics. The architecture is thus intrinsically insensitive to FSR mismatch in this single-mode regime. As illustrated in Fig. S10a, we show the transmission spectra of two resonators with an FSR difference of 100 MHz, where the central resonance modes are perfectly aligned at position ①. The corresponding Creutz ladder band structure is shown in Fig. S10b, demonstrating that such an FSR mismatch does not affect the simulated lattice when the central modes are aligned. This behavior contrasts with inter-mode synthetic frequency lattice schemes, where precise FSR matching is essential and even small mismatches can strongly modify the lattice structure and dynamics [7].

In our device, the measured FSRs of the two rings are 8.91 GHz and 8.89 GHz, corresponding to a mismatch of approximately 20 MHz. This value is well within the tolerance of the present scheme and has no observable impact on the experimental results. For completeness, we also indicate in Fig. S10a the cases where the photonic molecule resonances are detuned (positions ②, ③, and ④), for which the corresponding Creutz ladder band structures in Fig. S10b become progressively distorted as the detuning increases.

VII. ROBUSTNESS OF SSH LATTICE CONSTRUCTION TO FREQUENCY DETUNING

A. Regime where the modulation strength Is much smaller than the loss γ

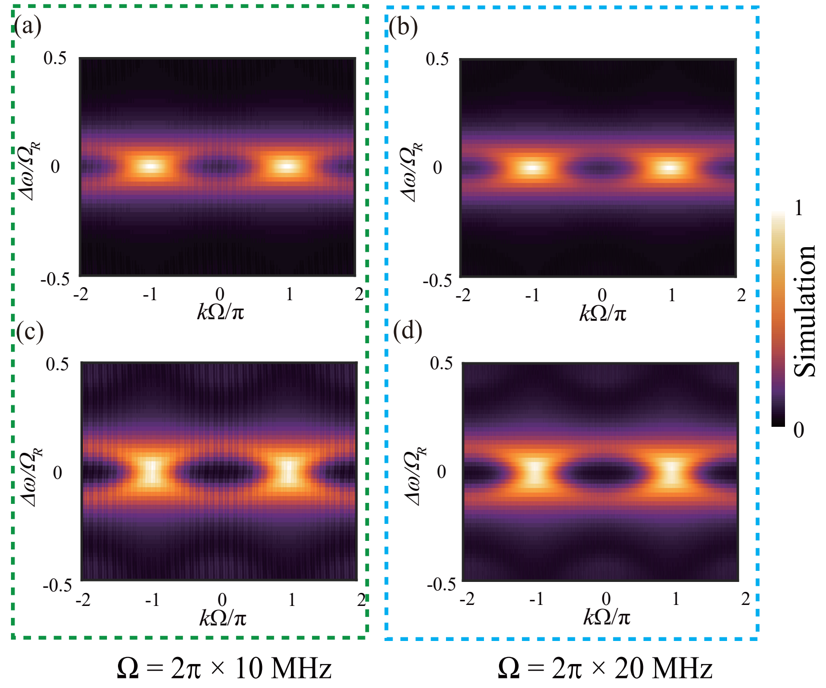

Fig. S11. **SSH band structure with different frequency separation between microwaves.** (**a, c**) Band structure of SSH lattice at RF modulations of $\Omega_1 = 2.5$ GHz and $\Omega_2 = 2.51$ GHz, with separation of Ω=10 MHz. (**b, d**) Band structure of SSH lattice at RF modulations of $\Omega_1 = 2.5$ GHz and $\Omega_2 = 2.52$ GHz, with Ω=20 MHz.

Leveraging the flexibility of construction of the intra-resonant frequency lattice, our SSH lattice implementation demonstrates robustness against the frequency detunings of the applied dual microwave signals. This robustness originates from the inherent nature of broadband and continuity of the intra-resonant frequency modes within the resonant peak, which loosens the condition of the lattice-site definition. Here, for simplicity, we consider the frequency separation between the dual microwave signals, which is related to both the detunings of these microwaves. As shown in Fig. S11, when the separation is 10 MHz, the measured band structure aligns well with the theoretical SSH model. Notably, with a 20-MHz separation, clear SSH band characteristics can still be observed. This result further highlights the significant advantage of our approach in achieving robust on-chip asymmetric coupling lattices, particularly in demonstrating tolerance to frequency detuning of the applied microwave signals.

B. Breakdown of the synthetic frequency lattice at large lattice constants

As shown in Fig. S12a, we extract the fitted full width at half maximum (FWHM) and the corresponding Q factor of the simulated resonant ring mode. In Fig. S12b, the resonance modes of the two rings are detuned by 2.5 GHz,

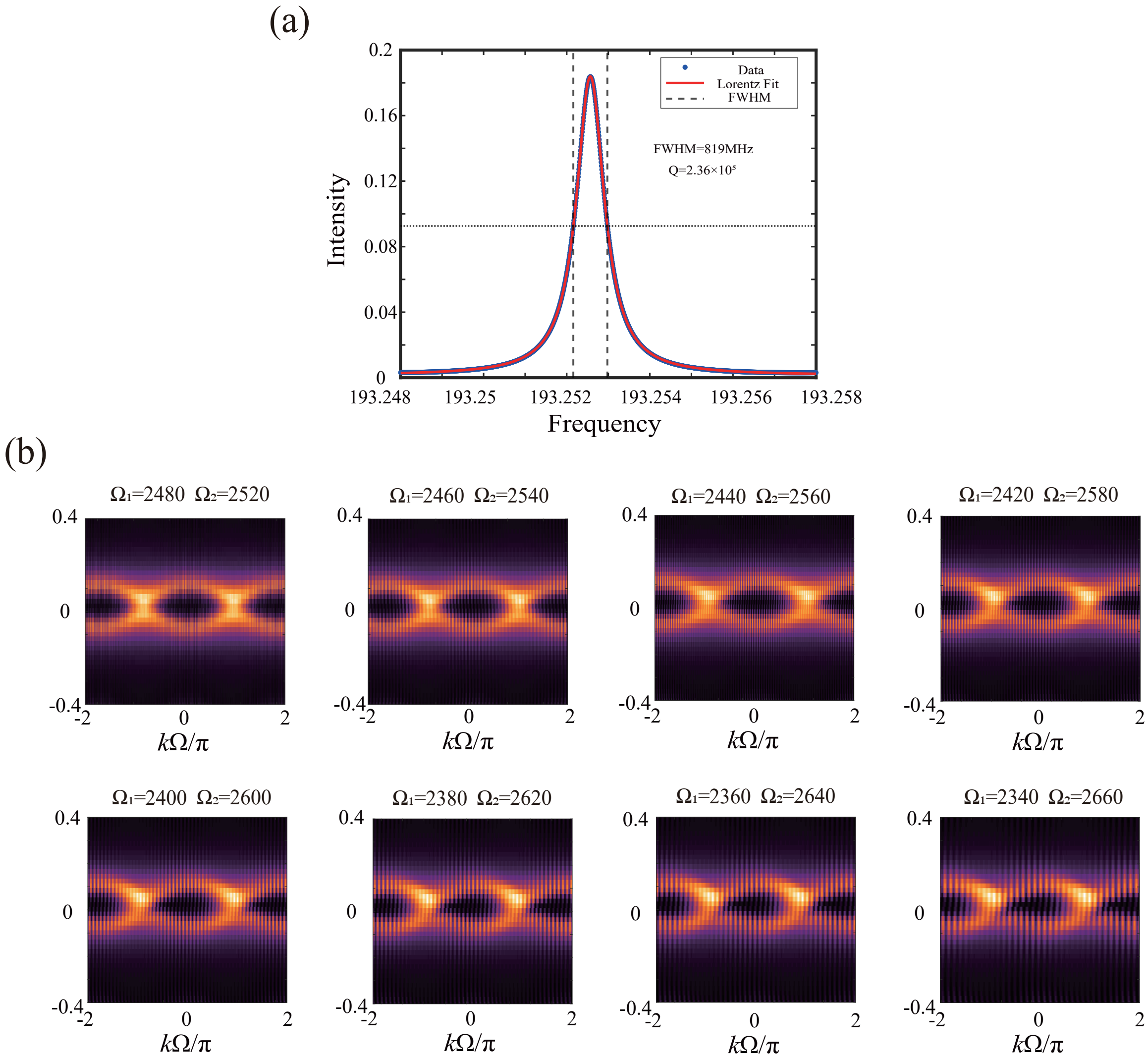

Fig. S12. **As the lattice constant of the synthetic frequency lattice increases, the model construction gradually breaks down.** (**a**) Fitted full width at half maximum (FWHM) of a single resonant mode. (**b**) Simulated band structures of the SSH model as the synthetic frequency lattice constant increases from 40 MHz to 320 MHz, showing progressive deformation and eventual breakdown of the model.

and the SSH model is constructed by applying two modulation tones to the MZI. As the frequency difference between the two modulation signals increases, the number of frequency lattice sites within a single resonant mode gradually decreases, leading to a progressive breakdown of the simulated SSH model. Specifically, as the modulation frequency difference is increased from 40 MHz to 320 MHz, the experimentally measured band structures increasingly deviate from the theoretical SSH band structure.

VIII. IMPACT OF RING RESONATOR LOSS AND RF MODULATION STRENGTH ON THE MEASURED BAND GAP OF THE SSH MODEL

For the SSH model in the topologically nontrivial regime, an bandgap is expected to open. In realistic experiments, however, the observability of this bandgap is strongly affected by band broadening induced by resonator loss, as well as by the ratio of the RF modulation strengths that determines the effective coupling contrast. To clearly illustrate the influence of these two factors, we perform systematic numerical simulations. As shown in Fig. S13, with a fixed

RF modulation strength ratio, reducing the resonator loss significantly suppresses band broadening and leads to a progressively more pronounced bandgap at the center of the spectrum. In contrast, Fig. S14 demonstrates that for a fixed resonator loss, increasing the RF modulation strength ratio results in a larger bandgap, thereby enhancing its visibility in experimental measurements. These results indicate that both low resonator loss and sufficiently large modulation contrast are crucial for clearly resolving the SSH bandgap in our platform.

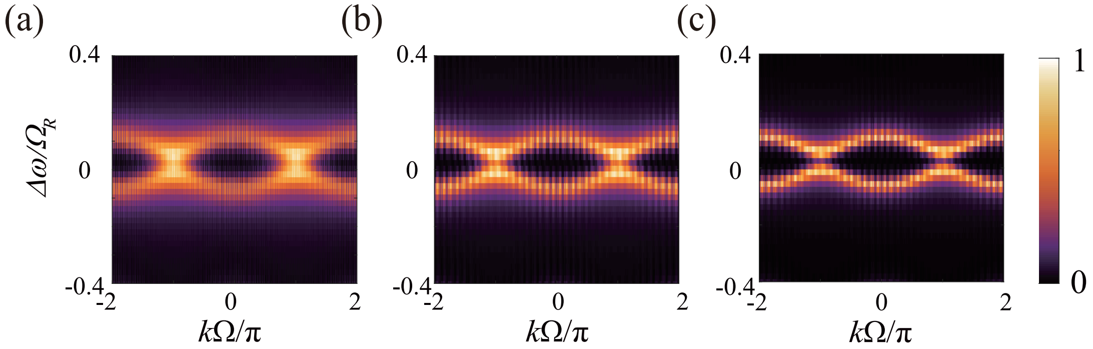

Fig. S13. **The influence of loss on the measurement of the band structure of the SSH model.** (**a-c**) The losses from left to right are 100, 50, and 10 dB/m, respectively, with a fixed RF modulation strength ratio of 1:2.

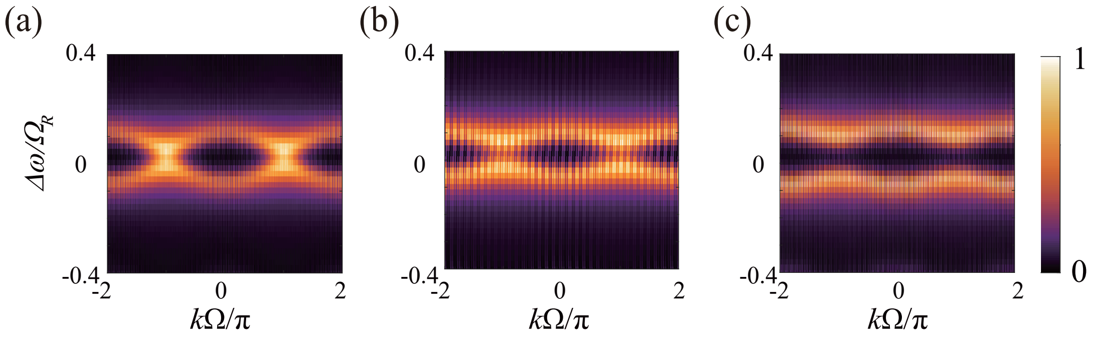

Fig. S14. **The influence of RF modulation strength ratio on the measured band structure of the SSH model.** (**a-c**) From left to right, the RF modulation strength ratios are $1:2$, $1:4$, and $1:6$, respectively.

IX. THE CONSTRUCTION OF THE EXTEND SSH MODEL

While the SSH Hamiltonian is chirally symmetric, adding an extra coupling between neighboring sites on the same sublattice breaks the chiral symmetry, but retains inversion symmetry of the SSH lattice to give the extended SSH (xSSH) lattice. Compared to the SSH model, the xSSH model exhibits more complex and richer topological behaviors. As shown in Fig. S15a, long-range coupling can be easily implemented in our system by simply applying the RF modulation with frequency Ω to the resonators. The xSSH model Hamiltonian in the k-space is:

$$H_k = \begin{bmatrix} 2J^H\cos(\Omega k) & J_1^C + J_2^C e^{i\Omega k} \\ J_1^C + J_2^C e^{-i\Omega k} & 2J^H\cos(\Omega k) \end{bmatrix}. \tag{S21}$$

By solving the momentum-space Hamiltonian of the xSSH model, the resulting band structure is shown in Fig. S15b. Figs. S15c-f show the band structure of the xSSH model obtained with numerical simulation which correspond to the theoretical results in Fig. S15b.

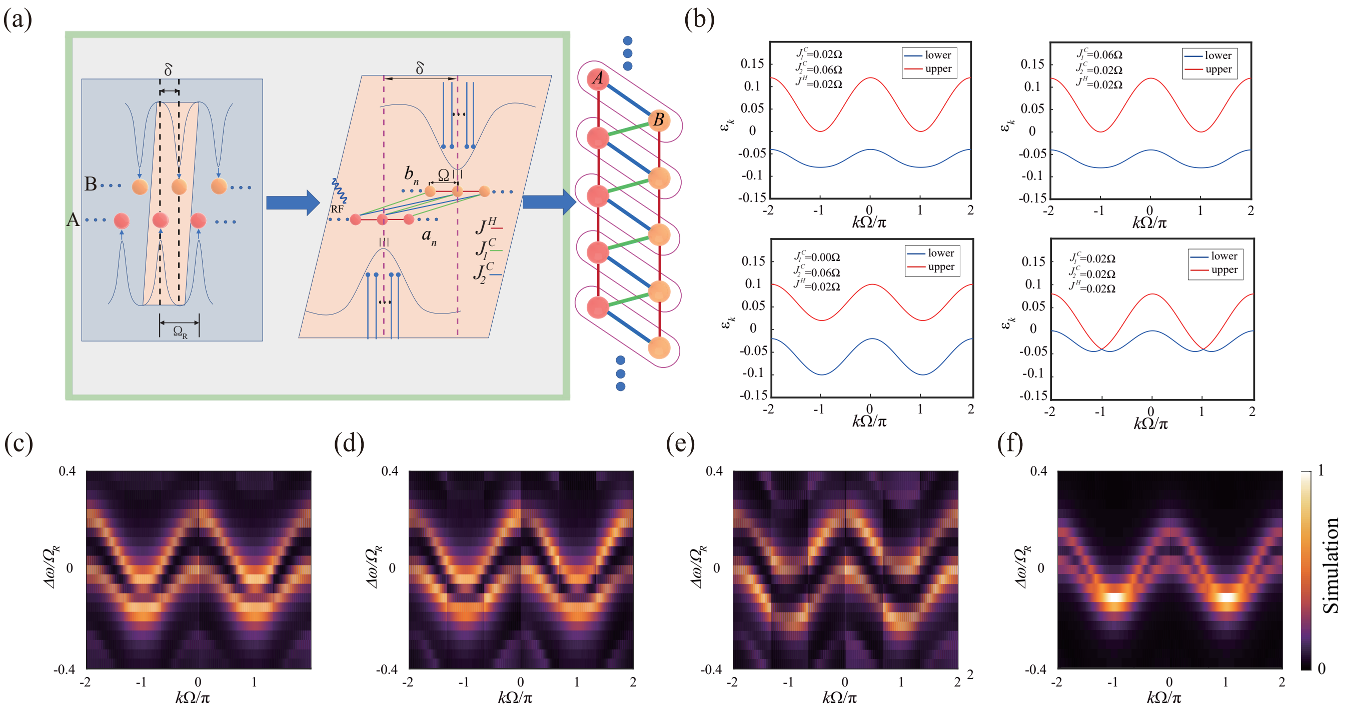

Fig. S15. **The band structure of the xSSH model.** (**a**) The resonant frequency peaks of Resonators A and B are detuned by δ using DC voltage. The J^H coupling is introduced by an RF modulation with frequency of Ω on the resonators. The simulation of xSSH model is realized using both the intra-resonant frequency-lattice sites and the inter-resonant sites. (**b**) Band structure corresponding to xSSH model, obtained by solving the k-space Hamiltonian. (**c-f**) Numerically-simulated solution of the xSSH model under different coupling parameters corresponding to those in Plot b.

X. QUALITY FACTOR OF THE RESONATORS

Figs. S16 and S17 illustrate the transmission signals from the drop ports of Resonators A and B in our MZI-assisted device respectively before and after the coating of the metal. The resonators exhibit a free spectral range (FSR) of approximately $\Omega_R = 2\pi \times 8.9$ GHz. Prior to metal deposition, the devices demonstrate a loaded quality factor of 3.7×10^5, which decreases to 1.7×10^5 after electrode fabrication due to absorption losses introduced by the metal.

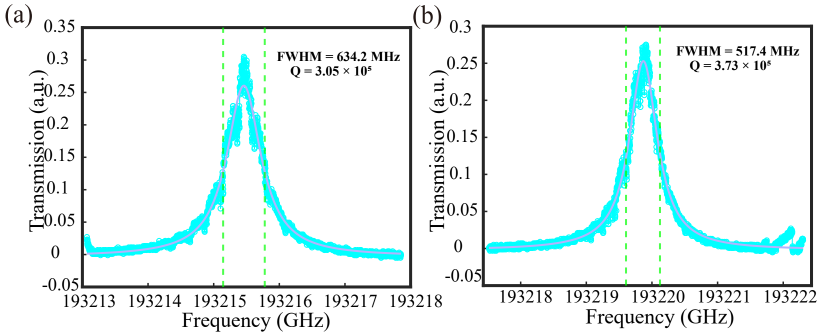

Fig. S16. **The transmission from the drop ports of Resonators A and B before the coating of the metal, respectively.**

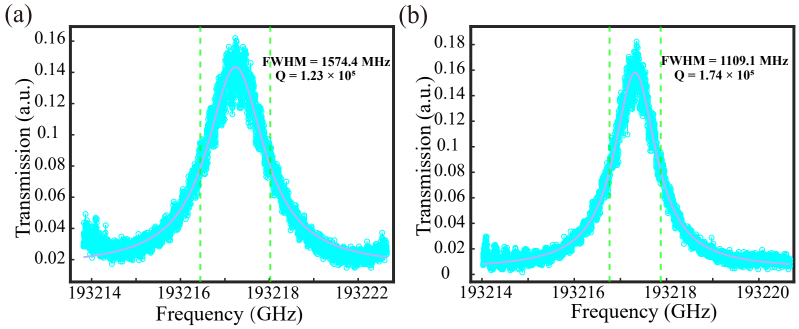

Fig. S17. **The transmission from the drop ports of Resonators A and B after the coating of the metal, respectively.**

[1] Z.-A. Wang *et al.*, On-chip photonic simulating band structures toward arbitrary-range coupled frequency lattices, Phys. Rev. Lett. **133**, 233805 (2024).

[2] Y. Hu, C. Reimer, A. Shams-Ansari, M. Zhang, and M. Loncar, Realization of high-dimensional frequency crystals in electro-optic microcombs, Optica **7**, 1189 (2020).

[3] I. Martin, G. Refael, and B. Halperin, Topological frequency conversion in strongly driven quantum systems, Phys. Rev. X **7**, 041008 (2017).

[4] Y. Song, T. Lei, Y. Xue, A. Cordaro, M. Haas, G. Huang, X. Li, S. Lu, L. Magalhaes, J. Yang, *et al.*, Universal dynamics and microwave control of programmable cavity electro-optic frequency combs, arXiv preprint arXiv:2507.21835 (2025).

[5] T. Lei, Y. Song, Y. Xue, Q. Gong, M. Lončar, and Y. Hu, Strong-coupling and high-bandwidth cavity electro-optic modulation for advanced pulse-comb synthesis, Light: Science & Applications **14**, 373 (2025).

[6] Y. Xue, X. Lv, G. Wu, T. Lei, C. Cao, Y. Lei, M. Wang, Y. Li, Q. Gong, D. Zhu, *et al.*, A universal framework for nonlinear frequency combs under electro-optic modulation, arXiv preprint arXiv:2511.21059 (2025).

[7] A. Dutt *et al.*, A single photonic cavity with two independent physical synthetic dimensions, Science **367**, 59 (2020).
